# Correction: Phenotypic and genotypic drug susceptibility patterns of *Mycobacterium tuberculosis* isolates from pulmonary tuberculosis patients in Central and Southern Ethiopia

**DOI:** 10.1371/journal.pone.0318279

**Published:** 2025-01-23

**Authors:** Melaku Tilahun, Teklu Wegayehu, Biniam Wondale, Tewodros Tariku Gebresilase, Tesfaye Gebreyohannes, Abraham Tekola, Mekdes Alemu, Sebsib Neway, Bethlehem Adnew, Maeruf Fetu Nassir, Yonas Kassahun, Abraham Aseffa, Kidist Bobosha

The fourth author’s name is spelled incorrectly. The correct name is: Tewodros Tariku Gebresilase. The correct citation is: Tilahun M, Wegayehu T, Wondale B, Gebresilase TT, Gebreyohannes T, Tekola A, et al. (2023) Phenotypic and genotypic drug susceptibility patterns of *Mycobacterium tuberculosis* isolates from pulmonary tuberculosis patients in Central and Southern Ethiopia. PLOS ONE 18(9): e0285063. https://doi.org/10.1371/journal.pone.0285063.
