# Supplementary material for: Phenotypic and genotypic drug susceptibility patterns of Mycobacterium tuberculosis isolates from pulmonary tuberculosis patients in Central and Southern Ethiopia
Source: PLoS One. 2023 Sep 8;18(9):e0285063. doi: 10.1371/journal.pone.0285063 (PMC10491001; doi:10.1371/journal.pone.0285063)
Supplement: S1 Table — (DOCX) [file pone.0285063.s001.docx]

**Table 1 Sociodemographic feature of the study participants in Central and Southern Ethiopia**

| Variables | Frequency, N (%) |
| --- | --- |
| Age Group | |
| 18 - 34 | 167(66.8) |
| 35 - 49 | 55(22.0) |
| 50^+^ | 28(11.2) |
| Gender | |
| Female | 91(36.4) |
| Male | 159(63.6) |
| Education | |
| Illiterate | 74(29.6) |
| Grade 1 - 8 | 104(41.6) |
| Higher Grade | 55(22.0) |
| Diploma and above | 17(6.8) |
| Occupation | |
| Civil Servant | 8(3.2) |
| Daily Laborer | 32(12.8) |
| Driver | 2(0.8) |
| Farmer | 67(26.8) |
| Merchant | 22(8.8) |
| NGO | 2(0.8) |
| Student | 56(22.4) |
| Unemployed | 30(12.0) |
| Unknown | 31(12.4) |
| NGO = Non-Government Organization | |
